# Coronary artery calcium before and after hospitalization with pneumonia: The MESA study

**DOI:** 10.1371/journal.pone.0191750

**Published:** 2018-02-08

**Authors:** Vicente F. Corrales-Medina, Girish Dwivedi, Monica Taljaard, William Petrcich, Joao A. Lima, Sachin Yende, Richard A. Kronmal, Julio A. Chirinos

**Affiliations:** 1 Department of Medicine, University of Ottawa. Ottawa, Ontario, Canada; 2 Clinical Epidemiology Program, The Ottawa Hospital Research Institute, Ottawa, Ontario, Canada; 3 Harry Perkins Institute of Medical Research and Fiona Stanley Hospital, The University of Western Australia, Perth, Australia; 4 Department of Cardiology, Johns Hopkins University, Baltimore, Maryland, United States of America; 5 The Clinical Research, Investigation, and Systems Modeling of Acute Illness (CRISMA) Center, Department of Critical Care Medicine, University of Pittsburgh, Pittsburgh, Pennsylvania, United States of America; 6 Department of Biostatistics, School of Public Health, University of Washington. Seattle, Washington, United States of America; 7 Division of Cardiology, University of Pennsylvania. Philadelphia, Pennsylvania, United States of America; University of Colorado Denver School of Medicine, UNITED STATES

## Abstract

**Background:**

Epidemiological analyses demonstrate that pneumonia survivors have a higher risk of myocardial infarction than people with similar load of risk factors for atherosclerotic cardiovascular disease (ASCVD) but without pneumonia. This may be due to a higher baseline burden of ASCVD in patients with pneumonia that is not captured by the accounting of known ASCVD risk factors in epidemiological analyses or to unfavorable accelerating effects of pneumonia on atherosclerosis.

**Methods:**

We analyzed data from the Multi-Ethnic Study of Atherosclerosis. We identified 54 participants that were hospitalized for pneumonia during study follow-up and that also had assessment of coronary artery calcium (CAC, an objective marker of coronary atherosclerotic burden) before and after this hospitalization. We matched them to 54 participants who were not hospitalized for pneumonia but that had CAC assessments at the same study visits as the pneumonia cases. We compared baseline CAC scores and their progression between groups.

**Results:**

Baseline CAC scores were similar in both groups (median [IQR]; 6.3 [0–356.8] in pneumonia participants vs. 10.8 [0–178.3] in controls; p = 0.25). After a median of 4.8 years, the direction and magnitude of CAC score change, and the slope of CAC score progression between groups was also similar (median change [IQR], 21.8 [0 to 287.29] in participants with pneumonia versus 15.8 [0 to 140.94] in controls, p = 0.28; difference in slope, 7.7, 95% CI -9.0 to 24.6, p = 0.18). However, among participants with high baseline ASCVD risk (i.e. ACC/AHA 10-year risk estimate ≥7.5%), participants with pneumonia showed a larger increase in CAC scores (median change [IQR]; 159.10 [38.55–407.34] versus 48.72 [0.97–246.99] in controls; p = 0.02) and a trend towards a steeper slope of CAC score progression (difference in slope, 19.7, 95% CI -6.6 to 45.6, p = 0.07).

**Conclusion:**

Pneumonia may accelerate the progression of atherosclerosis in people with high baseline ASCVD risk.

## Introduction

Pneumonia, together with influenza, is the eighth leading cause of death in the United States [1]. The overall rate of pneumonia among North American adults is approximately 5.16 to 6.11 cases per 1,000 person years and the yearly number of hospitalizations for pneumonia in the United States is estimated at 1.2 million [1, 2]. Likewise, atherosclerotic cardiovascular disease (ASCVD) is the principal cause of death worldwide as it is apparent from the burden of its three most important clinical manifestations: myocardial infarction (MI), stroke and peripheral arterial disease [3]. It is estimated that ASCVD affects over a third of the population in the United States [4].

Pneumonia is usually thought of as an acute process limited to the lungs with no lasting effects on the progression of chronic diseases in other organs. However, analyses of large cohorts suggest that pneumonia hastens the progression of atherosclerosis as patients that survive hospitalization for this infection have a subsequent increase in their risk of myocardial infarction (MI) both in the short (1 to 3-fold increase in the first year post infection) and long term (0.6 to1-fold increase after the first year post-infection) relative to controls without pneumonia [5, 6]. Given the prominence of pneumonia and ASCVD as causes of disease and death in humans [1–3], such association between pneumonia and ASCVD would have important clinical and public health implications.

Notwithstanding, in the analyses in which hospitalization with pneumonia was associated to a subsequent increase of MI risk [5, 6], adjustment for the baseline burden of ASCVD in pneumonia participants and controls was done indirectly using the prevalence ASCVD risk-factors as the surrogate. Because of this limitation, it has been proposed that the said association between hospitalization for pneumonia and increased risk of MI is only the reflection of a heavier baseline burden of ASCVD in pneumonia participants than is not captured by the load of ASCVD risk-factors that are measured in these analyses. An alternative explanation however, is that acute pneumonia indeed has accelerating effects on ASCVD even when the infection is successfully treated [7]. Exploring these possibilities was the aim of this study.

## Methods

We analyzed data from the Multi-Ethnic Study of Atherosclerosis (MESA), an observational cohort of 6,814 Americans ages between 45 and 84 years free of clinical ASCVD at study enrollment (between August, 2000 and July, 2002). MESA participants were recruited from six U.S. communities (Baltimore, MD; Chicago, IL; Forsyth County, NC; Los Angeles County, CA; northern Manhattan, NY; and St. Paul, MN). Sampling and recruitment procedures have been reported [8]. The MESA study was approved by the institutional review boards at all participating centers. All MESA participants provided written informed consent. The current study was approved by the Ottawa Health Science Network Research Ethic Board.

All MESA participants had assessment of their burden of coronary artery calcium (CAC; an objective marker of total coronary atherosclerotic burden) using computed tomography (CT) at study enrolment [9–12]. Thereafter, random subsets of participants had CAC assessments between July, 2002 and January, 2004 (2,953); January, 2004 and July, 2005 (2,805); and July, 2005 and July, 2007 (1,406). Finally, based on participation in a sub-study, 3,305 participants were evaluated for CAC between April, 2010 and February, 2012.

**Fig 1:**
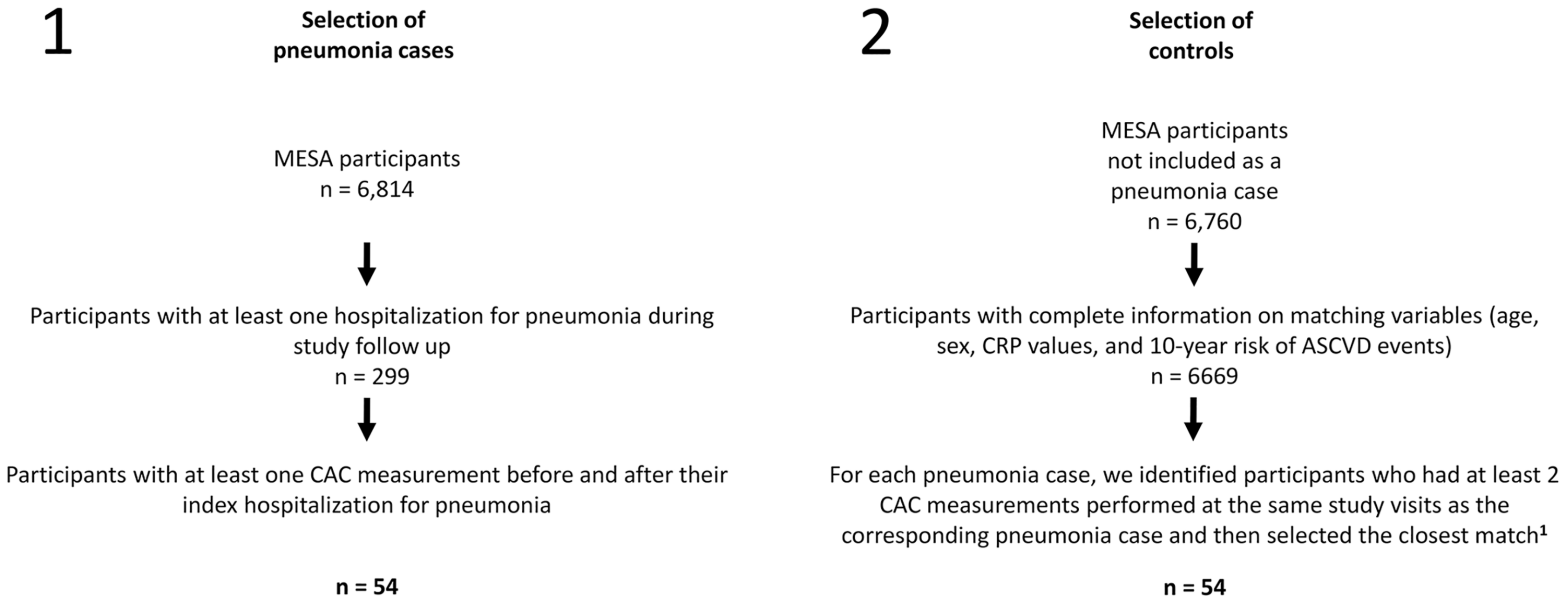
Selection of pneumonia cases and controls. Selection of pneumonia cases preceded the selection of controls. MESA denotes the Multi-Ethnic Study of Atherosclerosis. CAC denoted coronary artery calcium. CRP denotes serum C-reactive protein. ASCVD denotes atherosclerotic cardiovascular disease. ^**1**^ Matching was by age (±2.5 years), sex, CRP values (±0.5 mg/dL), and 10-year risk of ASCVD events (±2.5% as per the ACC/AHA ASCVD risk-equation). When there were multiple potential matches, we followed a greedy algorithm to select the closest match.

A diagram of the process that we followed for identification of our study subjects is presented in Fig 1. In MESA, all the hospitalizations of study participants that occurred during study follow-up were documented and their records retrieved. We identified hospitalizations with pneumonia during study follow-up by the presence of ICD-9 codes 481–486 in any of the first 5 discharge diagnoses fields of the discharge abstracts, as previously described [5, 13]. Henceforth, we identified 54 participants with at least one hospitalization with pneumonia and at least one CAC measurement before and after this hospitalization (if >1 hospitalization with pneumonia, we included only the first one and if ≥3 CAC measurements, we included only those immediately before and after this hospitalization). We matched each of these pneumonia cases to one control that also had serial CAC measurements at the same study visits as his corresponding pneumonia case but that was not hospitalized with this infection at any time before the second CAC evaluation. Matching was by age (±2.5 years), sex, CRP values (±0.5 mg/dL), and 10-year risk of ASCVD events (±2.5% as per the ACC/AHA ASCVD risk-equation) [14]. When there were multiple potential matches, we followed a greedy algorithm to select the closest match.

We compared baseline CAC scores and the magnitude of their change from the first to the second measurements between the groups using the Wilcoxon signed-rank test. We also compared the slope of CAC score change between groups using a linear mixed-effects regression model with fixed terms for pneumonia status, time (continuous variable), their interaction, and any baseline covariates that were statistically significantly different between the groups. A random subject effect accounted for correlation between matched-pairs and the heterogeneity in CAC baseline scores (intercepts) and their trajectories. We also conducted post-hoc subgroup analyses by the participants’ baseline 10-year risk of ASCVD events as per ACC/AHA guidelines (low risk, <7.5%; high risk, ≥7.5%) (14)]. As our hypotheses specifically implicated higher burden and steeper progression of CAC in participants with intervening pneumonia, we considered a one-tailed α<0.05 significant for all CAC comparisons between groups [15, 16]. We used SAS v9.3 (SAS Institute, Inc.; Cary, NC).

Our manuscript report conforms to the STROBE guidelines [17].

## Results

Demographic and clinical characteristics of the pneumonia and control groups are presented in Table 1. Other than a slightly higher HDL-cholesterol serum level in the group with intervening pneumonia (median [IQR], 54.5 [45.4 to 64.2] versus 47.5 [39.8 to 57.6] in controls; p = 0.02), all baseline ASCVD risk factors where similar between groups. Baseline CRP serum levels were also similar (median [IQR], 2.1 [1.0 to 3.7] in participants with pneumonia versus 2.2 [1.0 to 3.8] in controls; p = 0.93). The estimated median (IQR) baseline 10-year ASCVD risk in pneumonia participants and controls were 0.10 (0.05 to 0.18) and 0.10 (0.04 to 0.18), respectively (p = 0.98).

**Table 1:** Baseline characteristics of participants with intervening pneumonia in-between CAC measurements and matched controls without the infection.

Characteristics at the time of to the first CAC assessment	Participants hospitalized with pneumonia (n = 54)	Controls (n = 54)	P value
Age—mean (SD)	64.4 (8.4)	64.4 (8.6)	0.62
Male sex- no. (%)	26 (48.2)	26 (48.2)	1.00
White Caucasian race	29 (53.7)	23 (42.6)	0.26
10-year risk of ASVCD ^1^—median (IQR)	0.10 (0.05 to 0.18)	0.10 (0.04 to 0.18)	0.98
Diabetes—no. (%)	8 (14.8)	3 (5.6)	0.13
Total cholesterol (mg/dL)—mean (SD)	190.2 (41.1)	185.9 (28.4)	0.51
HDL cholesterol (mg/dL)—median (IQR)	54.5 (45.4 to 64.2)	47.5 (39.8 to 57.6)	0.02
Systolic blood pressure (mmHg)—mean (SD)	122.0 (19.3)	122.1 (19.4)	0.98
Diastolic blood pressure (mmHg)—mean (SD)	70.1 (7.9)	70.1 (9.7)	0.98
Body mass index—median (IQR)	28.8 (24.6 to 31.5)	27.7 (24.4 to 30.3)	0.38
CRP (mg/dL)—median (IQR)	2.1 (1.0 to 3.7)	2.2 (1.0 to 3.8)	0.93
On blood pressure treatment—no. (%)	23 (45.1)	25 (49.0)	0.68
Current Smoker—no. (%)	11 (21.2)	5 (9.6)	0.13
Baseline CAC score—median (IQR)	6.3 (0 to 356.8)	10.8 (0 to 178.3)	0.25

SD denotes standard deviation, IQR denotes inter-quartile range; ASCVD denotes atherosclerotic cardiovascular disease; CRP denotes C-reactive protein; CAC denotes coronary artery calcium.

^1^ As estimated by the ACC/AHA ASCVD risk-equation [14]

At the first CAC measurement, 22 (40.7%) pneumonia cases and 23 (42.6%) controls had CAC scores of 0. Overall, baseline CAC scores in both groups were not significantly different (median [IQR]; 6.3 [0 to 356.8] in the pneumonia group versus 10.8 [0 to 178.3] in controls, p = 0.25). The median (IQR) intervening times between CAC measurements were 4.8 years (3.1 to 6.7) and 4.9 years (3.0 to 6.5) in the pneumonia and control groups, respectively. For participants in the pneumonia group, the median (IQR) time between their intervening hospitalization for pneumonia and their second CAC measurement was 2.1 years (0.7 to 3.5). The direction and magnitude of CAC score change from the first to the second CAC measurements in both groups was similar (median [IQR]; 21.8 [0 to 287.3] in the pneumonia group versus 15.8 [0 to 140.9] in controls, p = 0.28). Concordantly, our adjusted linear mixed effect model revealed no statistically significant difference between the slope of CAC progression between groups (difference in slope 7.7, 95% CI -9.0 to 24.6, p = 0.18).

**Fig 2:**
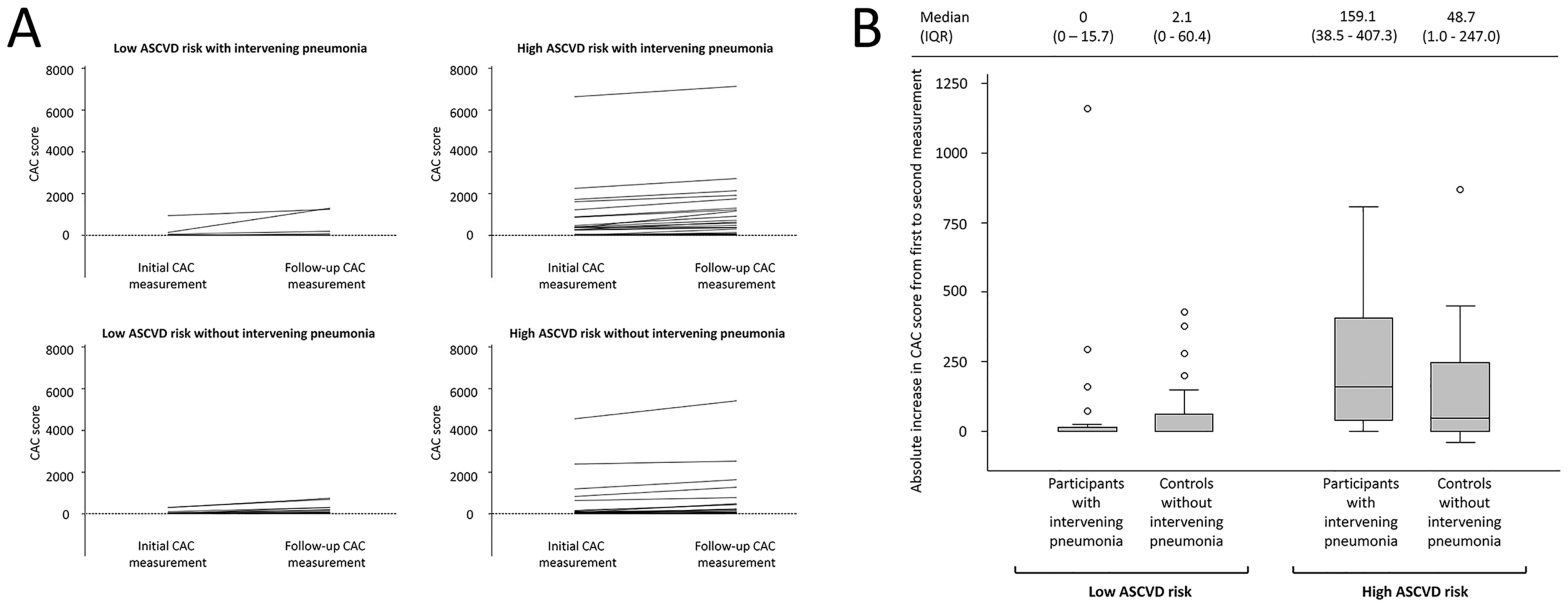
Trajectories of change (panel A) and absolute magnitude of change (panel B) of CAC scores in participants with and without intervening hospitalization with pneumonia stratified by their baseline risk of arteriosclerotic cardiovascular disease (ASCVD).

When participants were stratified by their estimated baseline 10-year risk of ASCVD, the magnitude of CAC score change among participants in the low-risk category (28 per group) remained similar between groups (median [IQR]; 0 [0 to 15.65] in pneumonia participants versus 2.07 [0 to 60.44] in controls; p = 0.25). However, among participants in the high ASCVD-risk category (26 per group, only 2 with a baseline CAC score of 0 per group), individuals with intervening pneumonia had a significantly larger increase in CAC scores (median [IQR]; 159.10 [38.55 to 407.34] versus 48.72 [0.97 to 246.99] in controls; p = 0.02) (Fig 2). Likewise, while the slope of CAC score progression among participants in the low ASCVD risk category was also similar between groups (difference in slope 0.8, 95% CI -19.5 to 21.1, p = 0.47), there was a trend towards a steeper slope of CAC progression in participants with intervening pneumonia compared to controls among participants in the high ASCVD-risk category (difference in slope 19.7, 95% CI -6.6 to 45.6, p = 0.07).

## Discussion

Participants with intervening pneumonia and controls had similar baseline burden of CAC after being matched by their overall burden of traditional ASCVD risk factors. This finding does not support the proposition that the previously reported association between hospitalization with pneumonia and subsequent MI-risk increase is spuriously driven by an unrecognized heavier baseline burden of ASCVD in pneumonia patients (relative to controls) that is not appropriately accounted for by adjustments based on the prevalence of ASCVD risk-factors in observational studies [5, 6]. Instead, this finding suggests that increments in MI-risk post-pneumonia would be related to biological post-infectious changes that accelerate atherosclerotic processes, promote thrombotic changes in blood, and/or increase the vulnerability of the myocardium to ischemia [18].

Regarding the possibility that pneumonia exerts accelerating effects on atherosclerosis, we found that CAC score progression was not significantly different between participants with intervening pneumonia and controls. An explanation for this finding could be the high proportion of participants with baseline CAC scores of 0 (i.e. their CAC load fell below the CT detection threshold, ~40% in both groups). Because such participants demonstrate very slow progression of CAC over several years [19, 20], it is possible that any effect of pneumonia on atherosclerosis progression was not detected in our analysis because of the inability of CT imaging to detect CAC progression in participants whose levels of CAC never reach the CT detection limit. Concordantly, when we stratified participants by their baseline ASCVD event risk, we observed that among participants in the higher ASCVD risk category (92% of whom had CAC scores >0 at baseline), participants with intervening pneumonia showed a trend towards a larger and steeper CAC progression than controls.

CAC is an objective marker of ASCVD burden and a robust independent predictor of future ASCVD events [9–12]. Within the current understanding of the ASCVD progression, coronary calcification is considered a late event distinctive of type 5 atherosclerotic lesions (fibroatheroma) [21]. Indeed, coronary calcification is thought to occur as a consequence of the instability and rupture, with subsequent calcification of type 4 lesions (formed atheroma) as part of their healing process [21]. Increased intra-plaque inflammation, especially in reference to the infiltration and activity of macrophages within atherosclerotic plaques, is thought to be pivotal for the progression of ASCVD including the complication and rupture of atherosclerotic lesions, and the calcification of vascular smooth muscle cells [22, 23]. Experiments in atherosclerosis-prone mice show that acute infections increase atheroma burden and the rate of macrophage infiltration in atherosclerotic plaques in the short and long-term after successful treatment of the infection [7]. Therefore, it is plausible that pneumonia can accelerate ASCVD progression (including coronary calcification) via the promotion of macrophage infiltration and activity within atherosclerotic plaques, an effect that, as suggested by our study, would be more evidently detected by the measurement of CAC progression if this measurement is performed in people with more advanced ASCVD at baseline (because coronary calcification is, as stated above, is a late event in ASCVD progression). Confirmation of such effect of pneumonia on ASVCD progression in future studies is important not only because of the public health dimensions of these two conditions (pneumonia and ASCVD) but also because it would support the study of atherosclerosis-disease-modifying interventions to reduce the risk of MI post-pneumonia.

In this study we relied on ICD-9 codes to ascertain hospitalizations with pneumonia, a method that while validated cannot rule out misclassification [5, 13]. Also, as our selection and matching processes resulted in a relatively small number of participants that were ultimately included in our analyses, our results should only be regarded as preliminary and further validation will be necessary.

## Conclusions

In conclusion, among adults with similar (and overall low) ASCVD risk, baseline burden and progression of CAC did not differ between participants with intervening pneumonia and controls without this condition; however, when only participants with high baseline ASCVD event risk were accounted for, there was a trend towards a larger and steeper progression of CAC in the pneumonia group. Further investigation of potential accelerating effects of pneumonia on atherosclerosis in high ASCVD-risk groups is warranted.
